# Clinicopathological Characteristics and Recurrence Patterns of Olfactory Neuroblastoma: A 15‐Year Tertiary Center Case Series

**DOI:** 10.1155/crot/4417318

**Published:** 2026-10-18

**Authors:** Mohsen Naraghi, Maryam Lotfi, Lindelwa Phakamile Mmema, Jazmin Kazravan, Amirhossein Babaei

**Affiliations:** ^1^ Department of Otorhinolaryngology-Head and Neck Surgery Tehran University of Medical Sciences Tehran Iran tums.ac.ir; ^2^ Orphans Worldwide Jacksonville Florida USA; ^3^ Department of Pathology Tehran University of Medical Sciences Tehran Iran tums.ac.ir; ^4^ Eswatini Antivenom Foundation Simunye Eswatini; ^5^ Mayo Clinic Jacksonville Florida USA mayo.edu; ^6^ Rhinology Research Society Tehran Iran

**Keywords:** chemoradiotherapy, esthesioneuroblastoma, Hyams grade, Kadish staging, olfactory neuroblastoma, recurrence, skull base surgery, surgical margins, surveillance

## Abstract

**Objectives:**

Olfactory neuroblastoma (ONB) is a rare malignant tumor of the sinonasal tract with heterogeneous clinical behavior and recurrence potential. Due to its low incidence, prognostic factors and optimal treatment strategies remain incompletely defined. The objective of this study was to describe the clinicopathological characteristics, treatment approaches, and recurrence patterns of ONB over a 15‐year period at a tertiary referral center.

**Methods:**

A retrospective case series and literature review were performed. Medical records of nine patients with histologically confirmed ONB treated between 2007 and 2022 were reviewed. Demographic characteristics, presenting symptoms, imaging findings, Hyams histologic grade, treatment modalities, and follow‐up outcomes were analyzed. Diagnosis in every case rested on characteristic morphology together with a full neuroendocrine immunohistochemical panel, and Hyams grade was assigned on the definitive resection specimen. Variables that were not uniformly documented across the 15‐year archive—clinical stage, surgical margin status and the anatomical compartment of recurrence—were deliberately not imputed and are reported as unavailable.

**Results:**

Patients ranged in age from 18 to 61 years (mean 41.3 ± 12.4 years; median 42 years), with a male predominance (6/9, 66.7%). The most common presenting symptoms were nasal congestion, headache, and nasal mass. All patients underwent surgical resection with curative intent, predominantly through endoscopic endonasal approaches, followed by adjuvant chemoradiotherapy. Within a uniform, protocol‐defined posttreatment observation window of 3 years (median follow‐up 36 months), tumor recurrence occurred in 4 of 9 patients (44.4%), and 5 of 9 patients (55.6%) remained free of documented recurrence. Recurrence was observed across different Hyams grades, including grade I tumors, without a statistically significant association between histologic grade, age, or gender and recurrence.

**Conclusions:**

ONB demonstrates variable clinical presentation and recurrence patterns, even following multimodal treatment. Histologic grade alone may not reliably predict recurrence in small cohorts. Recurrence despite low‐grade histology and uniformly administered chemoradiotherapy supports grade‐, stage‐, and margin‐adapted rather than uniform adjuvant strategies, and structured surveillance continued well beyond 5 years.

## 1. Introduction

Olfactory neuroblastoma (ONB), also known as esthesioneuroblastoma, is a rare and malignant tumor that originates from the olfactory epithelium, which lines the nasal cavity and paranasal sinuses [1]. This neoplasm was first described by Berger et al. in 1924 and continues to attract considerable clinical interest due to its uncommon nature and complex management [2]. ONB accounts for only 0.3% of all upper aerodigestive tract malignancies and about 2%–6% of all sinonasal malignancies. Its rarity often poses challenges in diagnosis and treatment [3].

ONB shows a slight male predominance. A bimodal age distribution with peaks in the second and in the fifth to sixth decades has traditionally been described, but this pattern was derived largely from small historical series. Contemporary population‐based analyses of the Surveillance, Epidemiology and End Results (SEER) registry—including 876 patients with a median age of 54 years, and an earlier cohort of 511 patients—instead demonstrate a unimodal distribution peaking in the fifth to sixth decades, with disease in patients younger than 20 years being distinctly uncommon [4]. The tumor nevertheless spans a wide age range and may behave more aggressively in younger patients [5]. The clinical symptoms of ONB can be nonspecific and often overlap with other sinonasal conditions, complicating early diagnosis. Commonly reported symptoms include nasal obstruction, epistaxis, hyposmia, and headache. In some cases, patients may also present with ophthalmological abnormalities due to the tumor’s proximity to the orbit [6]. These symptoms can be mistaken for more common conditions such as chronic rhinosinusitis, leading to delayed diagnosis.

The diagnosis of ONB is based on a combination of clinical features and histopathological findings. Imaging studies, including CT and MRI, are typically used to assess the extent of tumor involvement, particularly in the olfactory groove region [7]. The two cross‐sectional modalities are complementary rather than interchangeable: contrast‐enhanced CT best demonstrates bony erosion of the cribriform plate, fovea ethmoidalis and lamina papyracea, whereas gadolinium‐enhanced MRI is superior for defining dural, intracranial and orbital extension and for separating enhancing tumor from obstructed secretions. Both are therefore recommended in the pretreatment workup, supplemented where available by functional imaging [8].

Due to the rarity of ONB, the prognostic factors are poorly understood, and there is no universally accepted treatment protocol. The management of ONB usually involves a multidisciplinary approach, which includes surgery, radiotherapy, and chemotherapy. Surgery is the primary treatment method, aiming for complete removal of the tumor [9]. The choice of surgical approach depends on the tumor’s location, size, and the extent of involvement with surrounding structures. Postoperative radiotherapy is often employed to target any remaining tumor cells and to lower the risk of recurrence. Chemotherapy is generally reserved for cases of advanced or metastatic disease [10].

Given the limited regional data on ONB in Middle Eastern populations, this study aims to provide clinicopathologic insights and outcomes of ONB managed at a single tertiary center.

## 2. Material and Methods

### 2.1. Study Setting

This study is a case series based on a file review of patients at Amir Alam Hospital, Tehran, diagnosed with ONB and treated over 15 years, from 2007 to 2022. The objective was to examine the clinicopathological characteristics, treatment modalities, and outcomes of patients with ONB.

### 2.2. Study Population

The study population comprised all patients with a confirmed diagnosis of ONB according to Hyams’ grading system [11]. Microscopically, ONB typically presents as well‐defined nests or sheets of uniform cells, often separated by a richly vascular or hyalinized fibrous stroma (Figure 1A). Characteristic findings include the presence of pseudorosettes, particularly Homer–Wright rosettes, where neoplastic cells encircle a delicate fibrillar neural matrix (Figure 1B), along with interdigitating neuronal processes indicative of neuropil formation. Immunohistochemical profiling is essential, demonstrating diffuse staining for neuron‐specific enolase, synaptophysin, and chromogranin A, as well as markers such as CD56 (Figure 2). There is also variable reactivity of sustentacular cell S100 (Figure 3), which helps differentiate the tumor from other sinonasal pathologies, including sinonasal undifferentiated carcinoma (SNUC) and neuroendocrine carcinoma.

**FIGURE 1:**
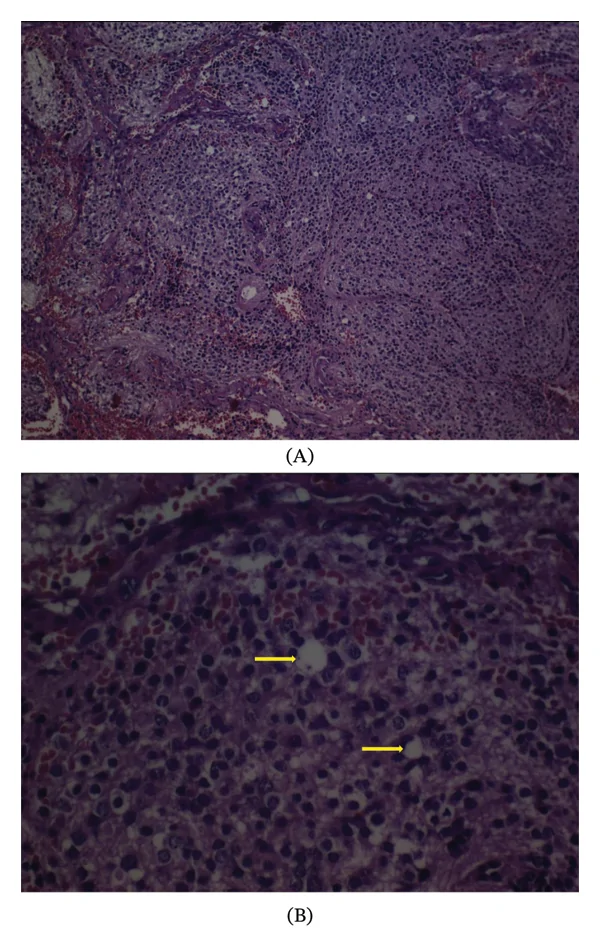
Microscopic examination of olfactory neuroblastoma reveals (A) uniform cell nests (H&E, ×100) and (B) prominent Homer–Wright rosettes (indicated by arrows), indicative of neuroblastic differentiation (H&E, ×400).

**FIGURE 2:**
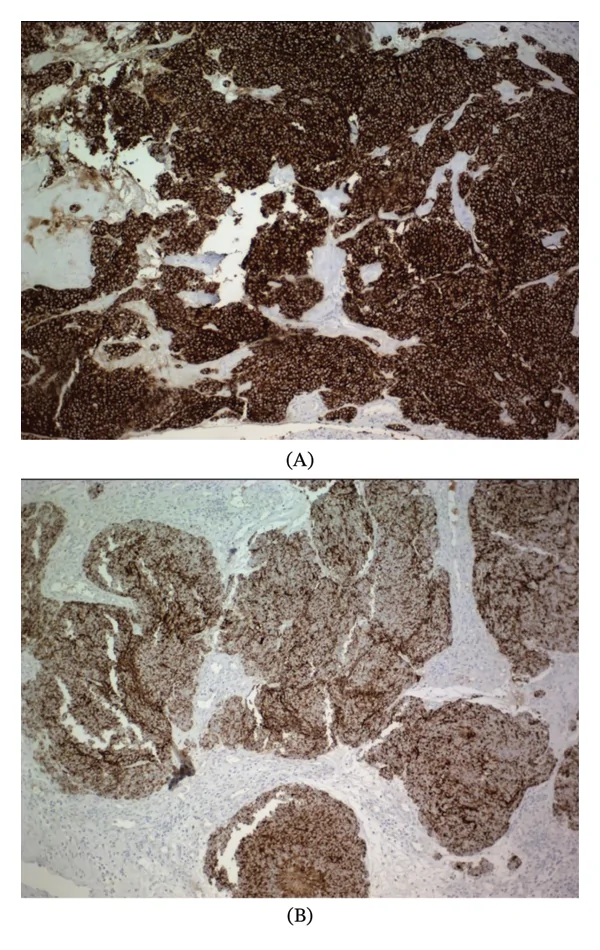
Immunohistochemical profiling reveals diffuse staining for (A) synaptophysin (×40) and (B) chromogranin A (×40).

**FIGURE 3:**
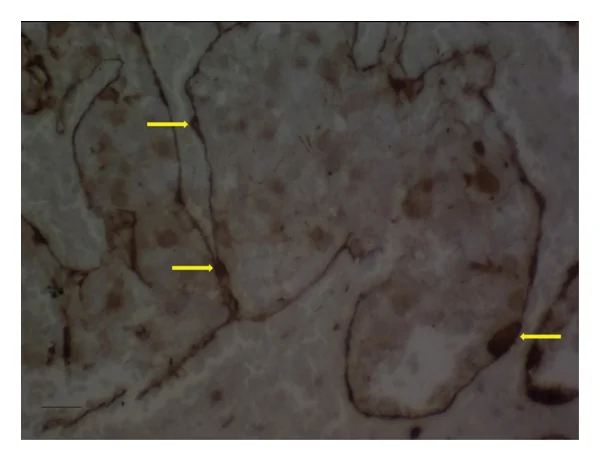
Sustentacular cells (indicated by arrows) are prominently located at the periphery of neuroblastoma cell nests, forming a distinct boundary that helps organize the tumor architecture (immunohistochemical profiling, ×400).

### 2.3. Pretreatment Workup, Staging and Grading

All patients were evaluated according to the institutional protocol for sinonasal malignancy, comprising complete otorhinolaryngological examination, rigid nasal endoscopy with tissue biopsy, cross‐sectional imaging of the sinonasal tract and anterior skull base with contrast‐enhanced computed tomography and/or gadolinium‐enhanced magnetic resonance imaging, and clinical assessment of the neck. Neither 18F‐FDG PET/CT nor somatostatin‐receptor‐directed 68Ga‐DOTATATE PET/CT was performed. Functional imaging was not routinely available or reimbursed for sinonasal malignancy at our institution during most of the study period, and SSTR‐directed imaging in particular has entered clinical practice only recently. This absence is acknowledged as a limitation of the pretreatment evaluation and is discussed further below. Diagnostic imaging was reviewed as part of clinical care and reported in the medical record; the original image archives for the earlier years of the series could not be retrieved in a form suitable for publication, and preoperative radiological figures are therefore not presented.

Histopathological diagnosis and Hyams grade were established on the definitive surgical resection specimen rather than on the diagnostic biopsy, since limited biopsy sampling is recognized to under‐represent the highest‐grade component of a heterogeneous tumor. Grading was not repeated after adjuvant therapy, and all grades reported here are therefore postoperative resection‐specimen grades. Cases were reviewed for the purposes of this study by the study pathologist to confirm the diagnosis and the assigned grade. It should be acknowledged that the Hyams system predates modern classification and endoscopic surgical practice and remains partly subjective: a recent detailed re‐grading study of 78 tumors reported frequent up‐grading when granular criteria were applied, with no tumor retaining a grade I designation [12].

Clinical staging systems for ONB ‐ the original and modified (Morita) Kadish systems and the Dulguerov modified TNM classification—were not uniformly documented in the archived clinical records across the entire 15‐year study period. Reliable retrospective assignment of these systems requires the original cross‐sectional imaging together with structured operative extent data, neither of which was retrievable for the full cohort, particularly for patients treated in the earlier years of the series. Rather than impute clinical stage from incomplete source data, and thereby introduce misclassification bias into a nine‐patient cohort, we elected to restrict the analysis to variables that could be verified for every patient. Hyams grade, assigned on the resection specimen and available for all nine patients, was accordingly used as the principal pathological stratification variable. The consequences of this decision, and the comparative prognostic performance of the Kadish, modified Kadish and Dulguerov systems, are addressed in the Discussion [8, 13].

### 2.4. Ethical Considerations

The study was conducted in accordance with the ethical guidelines set by the hospital’s review board (IR.TUMS.AMIRALAM.REC.1402.031). All patient data were anonymized to maintain confidentiality.

### 2.5. Data Collection

Data were collected from the patients’ medical records, which served as the primary source of information for the study. A standardized data extraction form was used to ensure consistency and accuracy in data collection. The following variables were extracted from each patient’s file:

• Demographic Information: Age at diagnosis and gender
• Presenting Symptoms: Symptoms at the time of initial presentation, including epistaxis, nasal obstruction, headache, and anosmia.
• Duration of symptoms
• Type of Surgery: The surgical approach used for tumor resection is categorized into the endoscopic endonasal approach or the craniofacial approach.
• Treatment Modalities: Details of adjunctive treatments, including chemoradiotherapy, radiotherapy, or chemotherapy alone, and their respective doses and regimens.
• Follow‐up and Outcomes: Posttreatment data included the 3‐year recurrence rate and any long‐term complications. Outcomes were accordingly assessed over a uniform 3‐year posttreatment observation window applied identically to every patient.

### 2.6. Surgical Management and Adjuvant Therapy

All nine patients underwent surgical resection with curative intent. Over the study period, the institutional approach shifted progressively from open craniofacial resection toward endoscopic endonasal skull base resection, and the endoscopic endonasal approach was the predominant technique in this cohort; the intraoperative appearance of en bloc removal of the cribriform plate is illustrated in Figure 4. Operative approach was, however, recorded in free‐text operative notes rather than in a structured data field, and for a subset of patients treated in the earlier years of the series the records did not permit unambiguous classification into a purely endoscopic versus a combined cranio‐endoscopic procedure. A patient‐level breakdown by approach is therefore not presented, since a table constructed from incompletely documented source data would convey false precision in a cohort of this size. For the same reason, surgical margin status ‐ which was not systematically stated in the histopathology reports of the earlier part of the series ‐ is not reported as a cohort variable; resection was undertaken with the intent of achieving negative margins.

**FIGURE 4:**
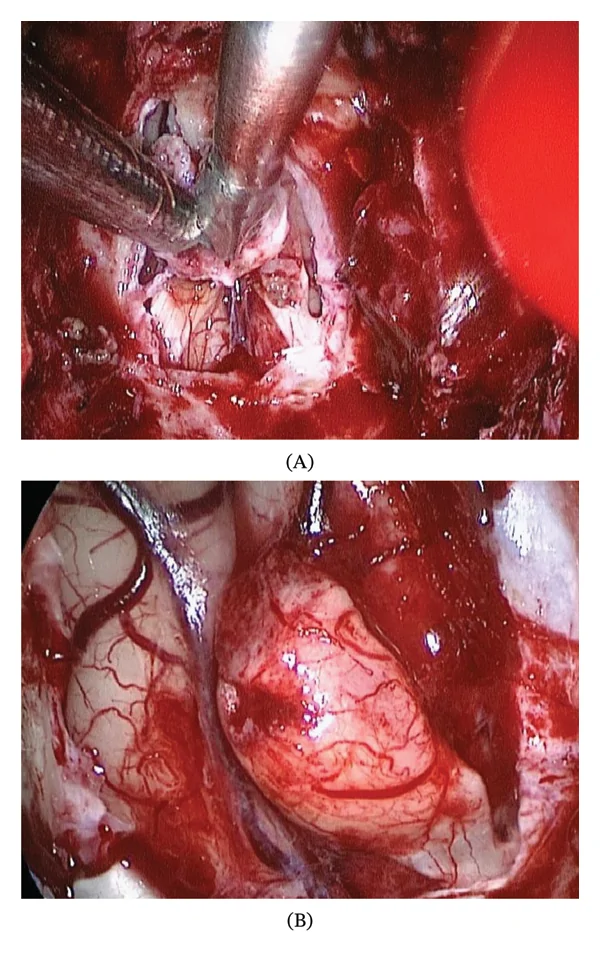
Endoscopic intraoperative view during resection of olfactory neuroblastoma. En bloc removal of the cribriform plate with progression toward the olfactory bulb (A), allowing direct visualization of the olfactory bulb (B).

All nine patients received adjuvant chemoradiotherapy. Adjuvant treatment was delivered at affiliated radiation‐oncology and medical‐oncology centers in line with contemporaneous institutional practice for sinonasal malignancy, comprising conventionally fractionated external‐beam radiotherapy to the tumor bed and surgical field together with platinum‐based systemic therapy. Individual prescribed doses, fractionation schedules, elective nodal coverage and the number of chemotherapy cycles were not consistently transcribed into the referring hospital record and could not be reconstructed retrospectively for every patient. Adjuvant therapy is therefore analyzed as a uniform binary exposure (received versus not received) rather than as a dose‐stratified variable. Contemporary dose recommendations, and the evidence bearing on selective rather than universal use of systemic therapy, are considered in the Discussion.

### 2.7. Follow‐Up Protocol and Outcome Definitions

After completion of treatment, patients were followed in the departmental skull base clinic. Surveillance comprised clinical review with rigid nasal endoscopy and clinical examination of the neck, supplemented by cross‐sectional imaging of the sinonasal tract and anterior skull base, at approximately three‐monthly intervals during the first two posttreatment years, at four‐to six‐monthly intervals thereafter, and annually beyond 5 years. This schedule is consistent with current recommendations for ONB, in which prolonged and effectively lifelong surveillance is advised because late recurrence is characteristic of this tumor, and in which regular nasal endoscopy, neck examination and interval MRI of the sinonasal tract and anterior skull base constitute the core of follow‐up, and in which chest imaging or PET/CT is reserved for higher‐risk disease or specific clinical indications [3, 6, 11]. That sentence describes the published surveillance standard against which our protocol is benchmarked; it does not describe investigations undertaken in this cohort. To remove any ambiguity on this point: No patient in the present series underwent PET/CT, which comprised initial staging and the whole of posttreatment surveillance up to and including the identification of recurrence. Surveillance was conducted by clinical review, rigid nasal endoscopy, clinical examination of the neck and anatomical cross‐sectional imaging (CT and/or MRI) alone. One consequence of the referral structure described above should be stated plainly at this point, because it governs much of what this report can and cannot say. Adjuvant treatment, and subsequently the investigation and management of recurrent disease, were delivered not at our institution but at the affiliated oncology centers to which patients were referred. The diagnostic workup undertaken once recurrence was suspected, any imaging performed at that stage, the salvage treatment given, and the subsequent clinical course were therefore documented in the case records of those centers, to which the investigators did not have access.

Recurrence was defined as radiologically and/or histologically documented disease occurring after a disease‐free interval following completion of primary treatment, and was recorded as a composite endpoint (any documented recurrence). The archived records did not consistently specify the anatomical compartment of recurrence—local, regional, or distant—nor the exact date on which recurrence was established.

### 2.8. Inclusion and Exclusion Criteria

The study included all patients diagnosed with ONB who underwent treatment at Amira’lam Hospital between 2007 and 2022. Patients were included regardless of gender. Only those with confirmed pathological results for ONB, based on histological examination and immunohistochemical staining, were considered eligible for inclusion.

Patients with incomplete medical records or missing critical data were excluded from the study to ensure comprehensive analysis.

### 2.9. Statistical Analysis

Descriptive statistics were used to summarize demographic information, clinical presentation, and treatment modalities. Continuous variables (e.g., age at diagnosis) were summarized as mean and standard deviation, while categorical variables (e.g., gender, symptoms, tumor grade, and treatment approaches) were expressed as frequencies and percentages. To evaluate factors associated with tumor recurrence, categorical variables were compared using the chi‐square test or Fisher’s exact test, as appropriate. For comparisons of continuous variables between recurrence groups, the independent samples *t*‐test was applied. Exact tests were preferentially reported when cell counts were limited. A *p* value < 0.05 was considered statistically significant. Statistical analyses were performed using SPSS software (IBM Corp., Armonk, NY, USA). Given the size of the cohort, all significance testing is regarded as exploratory and hypothesis‐generating rather than confirmatory: with nine patients and four events, the study has power to detect only very large effects, and nonsignificant results should not be read as evidence that an association is absent. Because vital status at a common census date could not be verified for the whole cohort and the exact date of recurrence was not uniformly recorded, time‐to‐event analysis was not feasible; Kaplan–Meier estimates of overall and disease‐free survival are therefore not reported, and outcome is expressed instead as the crude proportion of patients free of documented recurrence at last review.

## 3. Results

A retrospective review of medical records identified a total of 9 patients diagnosed with ONB.

### 3.1. Demographic and Clinical Characteristics

The study demonstrated a male predominance, with 6 male patients (66.7%) and 3 female patients (33.3%). The mean age at diagnosis was 41.3 ± 12.4 years, and the median age was 42 years, with a range of 18 to 61 years (Table 1). Eight of the nine patients (88.9%) were aged 33 years or older, and only one patient (11.1%) was younger than 20 years at diagnosis.

**TABLE 1:** Clinical data of the olfactory neuroblastoma.

Patient	Age	Gender	Presenting complaints	Duration of symptoms	Hyams grade	Recurrence
1	42	Female	Nasal mass	14 years	I	Yes
			Headache	3 months		
			Nasal congestion	3 months		

2	33	Male	Nasal congestion	6 months	II	No

3	18	Male	Nasal congestion	1 year	III	Yes
			Rhinorrhea	3 months		
			Sinusitis	1 year		
			Headache	1 year		
			Anosmia	6 months		

4	51	Male	Nasal pain	2 months	II	Yes
			Headache	2 years		
			Nasal congestion	2 years		

5	50	Female	Headache	2 years	II	No
			Seizure	2 years		

6	42	Male	Epistaxis	3 months	III	No
			Nasal mass	2 years		

7	61	Male	Nasal congestion	6 months	II	No
			Epistaxis	3 months		

8	34	Female	Nasal mass	1 year	I	No

9	41	Male	Nasal mass	2 years	I	Yes
			Headache	1 year		
			Nasal congestion	6 months		
			Nasal congestion	4 months		

### 3.2. Presenting Symptoms

The most common presenting symptom was nasal congestion, reported in 7 patients (30.4% of total reported symptoms), followed by headache in 5 patients (21.7%) and nasal mass in 4 patients (17.4%). Other presenting complaints included epistaxis (8.7%), sinusitis (4.3%), rhinorrhea (4.3%), anosmia (4.3%), nasal pain (4.3%), and seizure (4.3%). Clinical manifestations were heterogeneous and frequently nonspecific, which may contribute to delayed diagnosis. A detailed summary of presenting symptoms is provided in Table 2. Expressed per patient rather than per symptom entry, nasal congestion was reported by 6 of 9 patients (66.7%), headache by 5 of 9 (55.6%), nasal mass by 4 of 9 (44.4%), and epistaxis by 2 of 9 (22.2%); the frequencies given in Table 2 refer to individual symptom entries, one patient having recorded nasal congestion at two separate points during the prediagnostic interval. The interval between the onset of the earliest recorded symptom and diagnosis ranged from 2 months to 14 years, with a median of approximately 24 months.

**TABLE 2:** Frequency of symptoms.

Symptom	Frequency	Percent (%)
Nasal congestion	7	30.4
Headache	5	21.7
Nasal mass	4	17.4
Epistaxis	2	8.7
Sinusitis	1	4.3
Rhinorrhea	1	4.3
Anosmia	1	4.3
Nasal pain	1	4.3
Seizure	1	4.3

### 3.3. Treatment and Recurrence

All 9 patients underwent surgical resection of the tumor with curative intent, the endoscopic endonasal approach being the predominant surgical technique used across the study period (Figure 4). As detailed in the Methods, the archived operative records did not permit unambiguous patient‐level classification into purely endoscopic versus combined cranio‐endoscopic resection, and margin status was not systematically stated in the pathology reports of the earlier part of the series; neither variable is therefore reported as a cohort proportion. In addition, all 9 patients received adjuvant chemoradiotherapy as part of their treatment regimen.

Recurrence occurred in 4 of 9 patients (44.4%). With respect to gender, recurrence was observed in 3 of 6 males (50%) and 1 of 3 females (33.3%), with no significant association (*p* = 1.000). Similarly, Hyams grade did not demonstrate a significant relationship with recurrence (*p* = 0.714). Although numerically, grade I tumors showed recurrence in 2 of 3 cases (66.7%), compared to 1 of 4 (25%) in grade II and 1 of 2 (50%) in grade III, these differences were not statistically meaningful, likely due to the very small sample size and low expected cell counts. Age was also not significantly associated with recurrence; patients with recurrence had a mean age of 38.0 ± 14.1 years compared to 44.0 ± 11.7 years in those without recurrence (*p* = 0.507).

### 3.4. Outcome at Last Follow‐Up

Within the protocol‐defined 3‐year posttreatment observation window (median follow‐up 36 months), 5 of the 9 patients (55.6%) remained free of documented recurrence. Because the date on which recurrence was established and the vital status of every patient at a common census date could not be verified from the available records, formal Kaplan–Meier estimates of overall and disease‐free survival could not be generated; the recurrence‐free proportion reported here should therefore be read as a crude outcome measure rather than as an actuarial survival estimate, and it is presented with that caveat rather than omitted.

## 4. Discussion

ONB is a rare neuroectodermal malignancy originating from olfactory epithelium. The present 15‐year retrospective study provides valuable insights into the clinical and pathological features, treatment strategies, and outcomes associated with ONB. While the rarity of the disease poses significant challenges for large‐scale studies, our findings align with and add to the existing literature, particularly in terms of clinical presentation, recurrence rates, and therapeutic modalities.

Consistent with established epidemiological data, this study demonstrated a male predominance (66.7%), with a mean age at diagnosis of 41.3 ± 12.4 years (median 42 years) and an age range of 18 to 61 years. These findings mirror those reported by Lopez et al. (2024), who noted that ONB commonly affects individuals in their fifth to sixth decades, with a slight male bias [1]. Similarly, Ghanem et al. confirmed no strong gender or racial predilection but emphasized a peak incidence in the sixth decade [14].

The age structure of this cohort warrants specific comment, because a small series that does not reproduce the classically cited bimodal distribution may raise the question of whether other sinonasal small round blue cell tumors were inadvertently included. Three considerations argue against this. First, the diagnosis in every patient rested not on morphology alone but on the combination of characteristic architecture (well‐defined nests separated by a vascular or hyalinized fibrous stroma, with Homer–Wright pseudorosettes and intercellular neuropil) with a concordant immunohistochemical profile showing diffuse neuron‐specific enolase, synaptophysin, chromogranin A, and CD56 expression together with a peripheral sustentacular S100 pattern. That combination is not shared by SNUC, sinonasal neuroendocrine carcinoma, NUT carcinoma, rhabdomyosarcoma, lymphoma, mucosal melanoma, or Ewing sarcoma/peripheral primitive neuroectodermal tumor, each of which was considered and excluded, and cases were reviewed for this study by the study pathologist. Second, the bimodal distribution is itself increasingly regarded as an artefact of early, small, single‐institution reports: population‐based SEER analyses encompassing 876 and 511 patients have consistently demonstrated a unimodal distribution with a median age in the sixth decade, and pediatric and adolescent ONB is now recognized as distinctly rare [4]. Judged against that contemporary standard rather than against the historical bimodal model, a cohort with a median age of 42 years, eight of nine patients aged 33 years or older, and a single adolescent‐onset tumor is not anomalous. Third, the central tendency of a nine‐patient series is an inherently unstable statistic, and a single‐center cohort drawn from a population whose age pyramid is younger than that of the North American and European registry populations would be expected to sit below a registry median. We therefore interpret the observed age distribution as a consequence of sample size and referral population rather than of diagnostic heterogeneity.

Clinically, the most frequent presenting symptom in our study was nasal congestion (reported in 7 patients), followed by headache (5 patients) and nasal mass (4 patients). Less common symptoms included epistaxis, rhinorrhea, sinusitis, anosmia, nasal pain, and seizure (each observed in isolated cases). These manifestations are largely nonspecific and often overlap with benign sinonasal disorders, potentially contributing to delayed diagnosis. This diagnostic delay has also been described in the literature, with reported intervals between symptom onset and diagnosis ranging from 6 to 12 months [15]. Advanced disease may present with orbital or neurological manifestations; in our cohort, one patient presented with seizures, further illustrating the variable and sometimes atypical presentation of ONB.

Advanced imaging techniques, including CT and MRI, were used for tumor staging and to assess the involvement of adjacent structures such as the cribriform plate or orbit. These approaches are now further enhanced by PET/CT and emerging somatostatin receptor‐based imaging (e.g., 68Ga‐DOTATATE PET), which are particularly useful for detecting metastases and recurrent disease [16].

Neither 18F‐FDG PET/CT nor 68Ga‐DOTATATE PET/CT was available for the patients in this series, and this is a genuine limitation. 18F‐FDG PET/CT contributes principally to the detection of occult nodal and distant disease, which is material given that regional and distant spread ultimately develops in a substantial minority of patients, whereas somatostatin‐receptor‐directed imaging exploits the near‐universal expression of SSTR2 in ONB and is particularly useful in identifying small‐volume recurrent or metastatic deposits that are occult on anatomical imaging [16, 17]. The same receptor biology underpins peptide receptor radionuclide therapy (PRRT), so that SSTR‐directed imaging now functions as both a staging and a theranostic tool. We regard routine incorporation of functional imaging into pretreatment staging and posttreatment surveillance as the single most valuable upgrade to the diagnostic pathway for this disease, and recommend it wherever it is available.

A related limitation is that the Kadish, modified Kadish (Morita), and Dulguerov classifications could not be applied retrospectively with acceptable reliability, for the reasons set out in the Methods. Transparent omission was judged preferable to retrospective imputation, since misassignment of stage in a nine‐patient cohort would materially distort any apparent relationship between stage and outcome. The point nonetheless deserves emphasis, because clinical stage is prognostically informative. An individual participant data meta‐analysis of 399 patients staged with both systems found that increasing stage predicted worse disease‐free and overall survival under either scheme, that the Dulguerov system outperformed the Kadish system by information criterion, and that Kadish C is an outcome‐heterogeneous category which the Dulguerov T classification usefully subdivides [13]. A subsequent synthesis of large‐scale population‐based, multicenter and meta‐analytic evidence similarly confirmed Hyams grade, modified Kadish stage and the Dulguerov classification as independent prognostic factors, and identified surgery followed by radiotherapy as the strategy associated with the best overall and recurrence‐free survival in resectable disease [8]. On the basis of the present experience, we would advocate that Dulguerov T classification, modified Kadish stage, Hyams grade on the resection specimen, R status and the compartment of any recurrence be recorded contemporaneously as a minimum core dataset in every center managing this tumor.

Surgical margin status could likewise not be established for the cohort as a whole, and its prognostic weight is not in doubt. In the same individual participant data meta‐analysis, positive margin status predicted inferior disease‐free survival and unreported margin status behaved similarly to positive margin status [13]. We therefore decline to treat the absence of margin data as neutral, and interpret it conservatively as a plausible contributor to the 44.4% recurrence rate observed here, alongside the absence of stage‐adapted treatment selection discussed below.

All patients in our series underwent combined modality treatment consisting of endoscopic endonasal surgical resection followed by adjuvant chemoradiotherapy. Despite this aggressive multimodal approach, tumor recurrence occurred in 4 of 9 patients (44.4%). This recurrence rate falls within the range reported in the literature (approximately 30%–46% over long‐term follow‐up). Several studies, including Lopez et al., have emphasized the importance of complete surgical excision with negative margins and the role of adjuvant radiotherapy in reducing recurrence risk [1].

Endoscopic approaches have become increasingly preferred over traditional open techniques, as they offer lower morbidity and similar oncological control in selected cases. Our institution favored this approach in most patients, supporting the global trend toward minimally invasive surgery. Dharmarajan et al. [18] and Rodas et al. [19] advocate for unilateral resections with olfactory preservation in early‐stage, low‐grade tumors, reserving bilateral approaches for advanced cases.

However, the role of chemotherapy remains less well‐defined. It is primarily used for unresectable, metastatic, or recurrent ONB. As observed by Ghanem et al., the addition of chemotherapy may not significantly improve survival in the adjuvant setting and is best reserved for advanced‐stage cases [14]. The universal application of chemoradiotherapy in this study may have contributed to the observed recurrence rate of 44.4%. While chemoradiotherapy is often used in ONB management, several studies have suggested that stage‐adapted treatment (reserving chemotherapy for advanced or metastatic disease) may result in lower recurrence rates. For instance, Tajudeen et al. reported recurrence rates of less than 20% with tailored treatment approaches, suggesting that overtreatment or uniform application of chemotherapy may not necessarily yield improved outcomes [20]. Advanced Hyams grade (III) tumors, present in 22.2% of cases (2 of 9), likely contributed to recurrence, as high‐grade ONB is associated with occult metastasis and resistance to conventional therapies [11]. Read in this light, the present cohort functions as an unintended natural experiment. Because every patient received adjuvant chemoradiotherapy irrespective of grade or extent, the series describes outcomes under uniform rather than selective treatment, and the resulting recurrence rate of 44.4% sits at the upper rather than the lower end of the published range. This is concordant with the individual participant data meta‐analysis in which receipt of chemotherapy independently predicted worse disease‐free survival, a finding most plausibly explained by confounding by indication across the published literature, but one that is harder to attribute to selection alone in a cohort such as ours where systemic therapy was not indication‐driven [13]. We therefore read these data as real‐world support for grade‐, stage‐ and margin‐adapted adjuvant therapy: radiotherapy directed by grade, margin status and residual disease, with systemic therapy reserved for advanced, unresectable, recurrent or metastatic disease. Contemporary dose frameworks for the postoperative setting specify approximately 54 Gy after complete (R0) resection, 60 Gy for microscopically involved (R1) margins and 66–70 Gy for macroscopic residual (R2) disease, with definitive radiotherapy in the range of 60–70 Gy, and it is precisely the margin and stage information absent from our archive that would be required to apply such a framework prospectively [8, 11].

In our cohort, tumor recurrence was observed across different Hyams grades without a statistically significant association between histologic grade and recurrence. Recurrence occurred in 2 of 3 grade I tumors (66.7%), 1 of 4 grade II tumors (25%), and 1 of 2 grade III tumors (50%); however, these differences did not reach statistical significance (*p* = 0.714), likely due to the limited sample size. Although the literature generally supports an association between higher Hyams grades (III/IV) and poorer prognosis [11], our findings suggest that grading alone may not reliably predict recurrence in small cohorts. This observation aligns with more recent evidence indicating that histopathologic grading, particularly in the absence of detailed molecular characterization, may be insufficient as an isolated prognostic indicator [21]. Two further lines of evidence make this observation less anomalous than it may first appear. An individual participant data meta‐analysis consolidating the Hyams system concluded that the prognostic signal resides principally in the dichotomy between low (I‐II) and high (III‐IV) grade rather than in the four‐tier scheme. A dichotomy is too small to test, containing as it does only two high‐grade tumors. More strikingly, a 2025 re‐grading study of 78 tumors found that when granular, explicitly defined criteria were applied, cases were frequently upgraded and no tumor retained a grade I designation at all, and that neither original nor modified Hyams grade correlated significantly with outcome on univariable analysis over a median follow‐up exceeding 10 years [12]. That two of our three grade I tumors recurred is therefore consistent with an emerging recognition that a historical grade I designation should not by itself be used to justify de‐escalation of adjuvant therapy or abbreviation of surveillance.

Moreover, cervical nodal involvement and distant metastases were not documented at presentation in our study, although these occur in 11.5% [17] and 39% of patients [22], respectively, according to global data.

The anatomical pattern of recurrence could not be sub‐classified in the present cohort. The reason is structural rather than clerical: patients in whom recurrence was identified were referred onward for oncological management to the affiliated centers that had delivered their adjuvant treatment, and the records generated by that subsequent care remained in the custody of those institutions and were not accessible to the investigators. Our own records therefore document recurrence as an event without consistently specifying the compartment involved. This is a real limitation, because local, regional and distant failure carry materially different implications: local failure is frequently salvageable by revision endoscopic resection with or without re‐irradiation; regional failure remains the subject of continuing debate about elective neck irradiation, historical nodal failure rates of the order of 10% in electively untreated necks having been used to argue against routine prophylaxis, while more recent multi‐institutional data indicate that prophylactic neck irradiation significantly reduces nodal recurrence and that pathological dural invasion is itself associated with regional failure, respectively, according to global data [23].

In the absence of compartment‐specific documentation, we state only that recurrence occurred in four of nine patients within the protocol‐defined 3‐year observation window, and we deliberately refrain from reporting site‐specific proportions that cannot be verified from the source records.

The study by Lopez et al. offers a thorough review of histologic mimics and emphasizes the necessity for precise histopathologic interpretation to avoid misclassification, which has been an issue in earlier ONB studies [1]. Ghanem et al. extended this by showing that ONB may comprise molecularly distinct subgroups, some with high‐grade features and different responses to therapy [14].

Recent advancements in ONB research are reshaping therapeutic and diagnostic paradigms. Genomic profiling has identified frequent mutations in TP53, NF1, and CDKN2A, along with alterations in the PI3K/mTOR pathway. These mutations are associated with tumor aggressiveness and may serve as therapeutic targets in the future [24]. Furthermore, somatostatin receptor 2 (SSTR2) expression in nearly all ONB cases opens the door for PRRT using agents like 68Ga‐DOTATATE or octreotide analogs [17].

### 4.1. Contribution of the Present Series

We do not claim that this series overturns the established description of ONB. In a disease of this rarity, the value of a single‐center cohort lies in what it adds to an aggregate literature that is itself built almost entirely from small retrospective series, and several contributions may reasonably be claimed here. First, this is, to our knowledge, one of the few dedicated ONB series reported from Iran and among the very few from the wider Middle East, a region essentially unrepresented in the population‐based registries from which current prognostic models are derived; regional series are the only mechanism by which the transportability of those models to populations with different age structures and referral pathways can be examined, and the present cohort provides a data point that is otherwise absent from the literature. Second, the uniform application of surgery followed by adjuvant chemoradiotherapy to all nine patients, irrespective of grade or extent, offers an uncommon opportunity to observe outcomes under nonselective treatment; the resulting recurrence rate does not support the intuition that maximal uniform adjuvant therapy compensates for the absence of risk stratification, and this observation is directly relevant to centers that lack the staging infrastructure to individualize therapy. Third, recurrence in two of three Hyams grade I tumors adds a clinical observation to the emerging pathological evidence that historical grade I designations under‐represent biological risk, and argues concretely against using low grade alone to abbreviate surveillance. Fourth, the interval between first symptom and diagnosis extended to 14 years, with a median of approximately two years, well beyond the 6–12 months usually quoted, quantifying a diagnostic delay that is a health‐systems problem rather than a biological one and that is actionable through a low threshold for endoscopy and biopsy in unilateral, persistent, or otherwise unexplained sinonasal symptoms. Finally, the documentation deficits identified by this review are themselves generalizable to any center managing rare skull base tumors retrospectively, and the minimum core dataset proposed above is offered as a practical response to them.

This study’s main limitation is its retrospective nature and small sample size. Additionally, a lack of molecular profiling limits the ability to stratify patients fully. Prospective multicenter trials and the incorporation of genetic testing are essential to enhance understanding and optimize patient care. The reasons for recurrence were not documented in the medical records, and further studies are required to explore potential factors contributing to tumor recurrence, such as tumor size, surgical margins, and the adequacy of postoperative treatment. These limitations are stated here explicitly rather than left implicit. The study is retrospective and single‐center, and a cohort of nine patients with four events is too small to support multivariable modelling, so all comparisons are exploratory. Clinical stage (Kadish, modified Kadish and Dulguerov), surgical margin status, the anatomical compartment of recurrence, patient‐level operative approach and individual radiotherapy and chemotherapy dose data were not uniformly documented in an archive spanning 15 years; we have reported this transparently rather than imputing values that cannot be verified, and where such gaps plausibly influence interpretation—particularly margin status in relation to the observed recurrence rate—we have said so explicitly. Functional imaging with 18F‐FDG or 68Ga‐DOTATATE PET/CT was not performed, and preoperative image archives could not be retrieved in publication quality for the earlier years of the series. Vital status could not be confirmed for the entire cohort at a common census date, and time‐to‐event data were incomplete, so overall and disease‐free survival are not reported as actuarial estimates. Molecular characterization was not undertaken. Finally, a 3‐year observation window (median follow‐up 36 months) is short for a tumor in which recurrence beyond 5 years is well described, and the recurrence rate reported here should be regarded as a floor rather than a final figure. Taken together, these constraints define the agenda for the prospective, registry‐based and multicenter work that this disease requires, and they are the reason the present report is framed as a descriptive case series rather than as a prognostic study. Three points of methodological precision follow from the foregoing and are stated here so that they are not left to inference. First, the 3‐year figure reported throughout is a protocol‐defined observation window rather than an accrued mean follow‐up; the median follow‐up is accordingly 36 months, and freedom from recurrence at that point neither establishes cure nor supports inference about five‐ or 10‐year outcomes in a tumor with a well‐documented capacity for late relapse. Second, the published survival figures cited in the Results are contextual benchmarks only: because our endpoint is a crude proportion at a fixed time point and theirs are censoring‐adjusted actuarial estimates, no comparison of prognostic performance between this cohort and those series is intended, and none should be drawn. Third, no patient underwent functional imaging, so that occult regional or distant relapse may have gone undetected within the observation window. A fourth constraint underlies several of those already listed and is stated here in its own right. Adjuvant therapy and the management of recurrent disease were delivered at affiliated oncology centers rather than at our institution, and the clinical records generated by that care were not accessible to the investigators. This single feature of the referral pathway accounts for the absence from this report of individual radiotherapy and chemotherapy dose data, of the compartment and date of each recurrence, and of the salvage treatment and subsequent course of the four patients who relapsed. It is a limitation of the data available to us rather than of the care the patients received, and it is one that a prospective, shared‐record registry across the referral network would resolve; we regard the establishment of such a record as the most immediately actionable of the recommendations arising from this review.

## 5. Conclusion

This 15‐year case series highlights the complex clinical presentation and management of ONB. Our findings are consistent with global data regarding the disease’s epidemiology, recurrence rates, and treatment strategies. Despite aggressive multimodal treatment, recurrence continues to pose a challenge. Future efforts should focus on implementing routine molecular profiling and incorporating targeted and immunotherapeutic strategies to enhance patient outcomes in this rare malignancy. Three practical messages follow from this experience. Adjuvant therapy should be adapted to grade, stage, and margin status rather than applied uniformly. A low Hyams grade should not by itself license de‐escalated treatment or abbreviated follow‐up. Surveillance should be structured, endoscopic and imaging‐based, and should continue well beyond 5 years, given the propensity of this tumor to recur late. At a methodological level, the series also illustrates the cost of incomplete historical documentation in rare disease, and we would encourage centers managing ONB to record Dulguerov T classification, modified Kadish stage, Hyams grade on the resection specimen, R status, and the compartment of any recurrence as a routine minimum dataset. To that dataset, we would add the date of last contact and the date of any recurrence, recorded prospectively for every patient, since it is the absence of these two fields more than any other that prevented the present series from contributing actuarial survival data to the literature.

## Author Contributions

Mohsen Naraghi and Maryam Lotfi proposed the manuscript concept, took charge of the design, and gave final approval of the version.

Lindelwa Phakamile Mmema and Amirhossein Babaei performed data collection, drafted the article, and performed data analysis.

Jazmin Kazravan critically read and discussed the manuscript.

All authors discussed the results and implications and critical revision.

## Funding

This study received no external funding.

## Disclosure

No funding sources had any role in the study design; data collection, analysis, or interpretation; manuscript preparation; or the decision to submit the manuscript for publication. The lead author and manuscript guarantor, Mohsen Naraghi, affirms that this manuscript is an honest, accurate, and transparent account of the study being reported; that no important aspects of the study have been omitted; and that any discrepancies from the study as planned (and, if relevant, registered) have been explained.

All authors have read and approved the final version of the manuscript. The corresponding author, Mohsen Naraghi, had full access to all of the data in this study and takes complete responsibility for the integrity of the data and the accuracy of the data analysis.

## Ethics Statement

The study was conducted in accordance with the ethical guidelines set by the hospital’s review board (IR.TUMS.AMIRALAM.REC.1402.031). All patient data were anonymized to maintain confidentiality.

## Consent

Written informed consent was obtained from all participants (or their legal guardians) prior to inclusion in the study, in accordance with institutional and ethical guidelines.

## Conflicts of Interest

The authors declare no conflicts of interest.

## Data Availability

SPSS syntax and a minimal data dictionary enabling reproduction of the reported tables are available from the corresponding author upon reasonable request. The data supporting the findings of this study are available from the corresponding author upon reasonable request.
